# Regulation of MEK inhibitor selumetinib sensitivity by AKT phosphorylation in the novel *BRAF* L525R mutant

**DOI:** 10.1007/s10147-023-02318-w

**Published:** 2023-03-01

**Authors:** Chikako Nakai, Sachiyo Mimaki, Koutatsu Matsushima, Eiji Shinozaki, Kentaro Yamazaki, Kei Muro, Kensei Yamaguchi, Tomohiro Nishina, Satoshi Yuki, Kohei Shitara, Hideaki Bando, Yutaka Suzuki, Kiwamu Akagi, Shogo Nomura, Satoshi Fujii, Masaya Sugiyama, Nao Nishida, Masashi Mizokami, Yasuhiro Koh, Takuya Koshizaka, Hideki Okada, Yukiko Abe, Atsushi Ohtsu, Takayuki Yoshino, Katsuya Tsuchihara

**Affiliations:** 1 Division of Translational Informatics, Exploratory Oncology Research and Clinical Trial Center, National Cancer Center, 6-5-1 Kashiwanoha, Kashiwa, Chiba 277-8577 Japan; 2 G&G Science Co. Ltd., 4-1-1 Misato, Matsukawamachi, Fukushima 960-1242 Japan; 3 Department of Gastroenterological Chemotherapy, Cancer Institute Hospital of Japanese Foundation for Cancer Research, 3-8-31 Ariake, Koto-ku, Tokyo, 135-0063 Japan; 4 Division of Gastrointestinal Oncology, Shizuoka Cancer Center, 1007 Shimo-Nagakubo, Nagaizumi-Cho, Sunto, Shizuoka 411-8777 Japan; 5 Department of Clinical Oncology, Aichi Cancer Center Hospital, 1-1 Kanokoden, Chikusa-ku, Nagoya, 464-8681 Japan; 6 Department of Gastrointestinal Medical Oncology, National Hospital Organization Shikoku Cancer Center, 160 Minamiumemotomachi, Matsuyama, Ehime 791-0245 Japan; 7 Department of Gastroenterology and Hepatology, Hokkaido University Hospital, Sapporo, Japan; 8 Department of Gastroenterology and Gastrointestinal Oncology, National Cancer Center Hospital East, 6-5-1 Kashiwanoha, Kashiwa, Chiba 277-8577 Japan; 9 Department of Computational Biology, Graduate School of Frontier Sciences, The University of Tokyo, 5-1-5 Kashiwanoha, Kashiwa, Chiba 277-8561 Japan; 10 Division of Molecular Diagnosis and Cancer Prevention, Saitama Cancer Center, 818 Komuro, Inami-machi, Kitaadachi, Saitama 362-0806 Japan; 11 Biostatistics Division, Center for Research and Administration and Support, National Cancer Center, 6-5-1 Kashiwanoha, Kashiwa, Chiba 277-8577 Japan; 12 Department of Molecular Pathology, Yokohama City University School of Medicine, 3-9 Fukuura, Kanazawa-ku, Yokohama, Kanagawa 236-0004 Japan; 13 Genome Medical Sciences Project, National Center for Global Health and Medicine, 1-7-1 Kohnodai, Ichikawa, Chiba 272-8516 Japan; 14 Third Department of Internal Medicine, Wakayama Medical University, 811-1 Kimiidera, Wakayama, 641-8509 Japan; 15 National Cancer Center Hospital East, 6-5-1 Kashiwanoha, Kashiwa, Chiba 277-8577 Japan

**Keywords:** *BRAF* L525R, Extracellular signal-regulated kinase, Selumetinib

## Abstract

**Background:**

Oncogenic mutations in *BRAF* genes are found in approximately 5–10% of colorectal cancers. The majority of *BRAF* mutations are located within exons 11–15 of the catalytic kinase domains, with *BRAF* V600E accounting for more than 80% of the observed *BRAF* mutations. Sensitivity to BRAF- and mitogen-activated protein kinase (MEK) inhibitors varies depending on *BRAF* mutations and tumor cell types. Previously, we newly identified, *BRAF* L525R-mutation, in the activation segment of the kinase in colorectal cancer patient. Here, we characterized the function of the *BRAF* L525R mutation.

**Methods:**

HEK293 cells harboring a *BRAF* mutation (V600E or L525R) were first characterized and then treated with cetuximab, dabrafenib, and selumetinib. Cell viability was measured using WST-1 assay and the expression of proteins involved in the extracellular signal-regulated kinase (ERK) and protein kinase B (AKT) signaling pathways was evaluated using western blot analysis.

**Results:**

The MEK inhibitor selumetinib effectively inhibited cell proliferation and ERK phosphorylation in *BRAF* L525R cells but not in *BRAF* V600E cells. Further studies revealed that AKT phosphorylation was reduced by selumetinib in *BRAF* L525R cells but not in *BRAF* V600E cells or selumetinib-resistant *BRAF* L525R cells. Moreover, the AKT inhibitor overcame the selumetinib resistance.

**Conclusions:**

We established a model system harboring *BRAF* L525R using HEK293 cells. *BRAF* L525R constitutively activated ERK. AKT phosphorylation caused sensitivity and resistance to selumetinib. Our results suggest that a comprehensive network analysis may provide insights to identify effective therapies.

**Supplementary Information:**

The online version contains supplementary material available at 10.1007/s10147-023-02318-w.

## Introduction

BRAF is a serine-threonine kinase that acts downstream of the epidermal growth factor (EGF) receptor (EGFR) in the rapidly accelerated fibrosarcoma (RAF)/mitogen-activated protein kinase (MEK)/extracellular signal-regulated kinase (ERK) kinase pathway [1]. The RAF/MEK/ERK pathway has attracted much attention in the search for novel chemotherapeutic agents since mutations in *BRAF* are found in approximately 60% of melanoma [2, 3], 35–50% of papillary thyroid cancers [4, 5], 35% of low-grade ovarian serous tumors [6, 7], 5–10% of colorectal cancers (CRC) [8–10], and 5% of non-small cell lung cancers [11]. Genomic analyses have revealed various *BRAF* mutations in cancers, most of which occur in the kinase domain of the enzyme [12]. More than 80% of *BRAF* mutations include T to A transversions at nucleotide 1799, resulting in a substitution of valine (V) with glutamic acid (E) at codon 600 (V600E) in exon 15. Subtypes of *BRAF* mutations are classified as high, intermediate, and impaired depending on their effect on the kinase activity [13]. V600E causes constitutive activation of the downstream effectors MEK and ERK [14]. Another activation segment mutant, L597V, and two glycine-rich loop mutants, G464V and G469A, also enhance MEK/ERK signaling [2, 13]. In contrast, the *BRAF* G469E mutation significantly decreases MEK/ERK signaling. Meanwhile, other *BRAF* mutations, such as R462I, I463S, and G464E, do not increase MEK/ERK signaling [13, 15].

Although several *BRAF* mutations have been identified, the biological effects of these mutations are not fully understood [2, 13, 16, 17]. In a previous study using comprehensive genome-wide sequencing, we identified some non-V600E *BRAF* mutations in specimens from metastatic CRC (mCRC) patients with anti-EGFR antibody resistance [18]. Among the newly identified *BRAF* mutations, Q524L and L525R in the activation segment and L525R lead to enhanced kinase activity and increased ERK phosphorylation, but Q524L demonstrates similar activity as wild-type (WT) BRAF in transiently expressed cells [18]. We hypothesized that L525R may contribute to primary resistance to cetuximab, consistent with the lack of response to anti-EGFR antibody treatment.

Several preclinical and clinical studies have demonstrated that MAPK pathway inhibitors, such as inhibitors of BRAF and MEK, exhibit antitumor activity in cancers with *BRAF* mutations [19–21]. However, the use of these inhibitors as monotherapy frequently results in the development of resistance. Combination therapy using both BRAF and MEK inhibitors can improve overall survival for patients with *BRAF*-mutant melanoma [22–24]. Moreover, accumulating evidence suggests that the combination of an MEK inhibitor, such as selumetinib, and a phosphoinositide 3-kinase (PI3K)/protein kinase B (AKT) inhibitor can be effective in many cancer therapies [25]. In the current study, we functionally analyzed the *BRAF* L525R mutation in a stable cell line. We also evaluated the PI3K/mechanistic target of rapamycin (mTOR) inhibitor for inhibiting the proliferation of selumetinib-resistant *BRAF* L525R cells. We hypothesize that the PI3K/AKT signaling pathway underlies the drug resistance observed in these cells and propose the use of combination therapies.

## Materials and methods

### Chemicals and antibodies

Cetuximab (ERBITUX^®^) was purchased from Merck Serono Co., Inc. (Darmstadt, Germany) and dabrafenib was purchased from Adoq Bioscience (Irvine, CA, USA). Selumetinib and BEZ235 were purchased from Selleck Chemicals (Houston, TX, USA). Anti-EGFR (D38B1) XP, anti-phospho-EGFR (Tyr1068), anti-p42/44, anti-Pp42/44 (T202/Y204), anti-AKT (pan), anti-pAKT (S437), anti-Flag, and anti-GAPDH antibodies were purchased from Cell Signaling Technology, Inc. (Danvers, MA, USA).

### Cell culture and transfection

Human embryonic kidney 293 (HEK293) cells expressing WT EGFR [18] were cultured at 37 °C under 5% CO_2_ in Dulbecco’s Modified Eagle’s Medium supplemented with 10% fetal bovine serum. Flag-tagged *BRAF* V600E and *BRAF* L525R constructs were generated by performing site-directed mutagenesis of flag-tagged WT clones using the Prime STAR Mutagenesis Basal Kit (Takara Bio Inc., Shiga. Japan) by following the manufacturer’s instructions. Stable cell clones co-transfected with WT *EGFR* and *BRAF* were obtained by transfection of cells using FuGENE HD (Promega Corporation, Madison, WI, USA) followed by puromycin selection. All clones were confirmed by sequencing. The selumetinib-resistant L525R (L525R-R) cells were established by growing the L525R clone in the presence of 10 µM selumetinib for 2 months. The surviving cells at that point were considered selumetinib-resistant and maintained in the presence of 20 µM of selumetinib.

### Cell viability

Cells were seeded in 96-well culture plates at approximately 1 × 10^4^ cells/well and treated with cetuximab (0.05–50 µg/mL), dabrafenib (0.01–10 µM), selumetinib (0.01–10 µM), or BEZ235 (0.01–1 µM) for 72 h. Cell viability was assessed using Cell Counting Kit-8 (CCK8; Dojindo Laboratories, Kumamoto, Japan). The optical density of the cell culture medium in each well was measured at 450 nm using a microplate reader (Molecular Devices, San Jose, CA, USA). Three independent experiments were performed in triplicate for each of the drug concentrations.

### Western blotting

Cells were lysed in a buffer containing 50 mM Tris (pH 7.5), 150 mM NaCl, 1% NP-40, 0.25% sodium deoxycholate, 5 mM EDTA (pH 8.0), 50 mM NaF, 1 mM Na_3_VO_4_, and a commercial protease inhibitor cocktail (P8340) (Sigma-Aldrich Co. LLC, St. Louis, MO, USA). Protein concentration of the cell lysates was determined using a bicinchoninic acid (BCA) protein assay (Thermo Fisher Scientific, Waltham, MA, USA). Whole cell lysates were separated on a 4–20% gradient sodium dodecyl sulfate–polyacrylamide gel (FUJIFILM Wako Pure Chemicals, Osaka, Japan) and transferred onto polyvinylidene fluoride membranes (Bio-Rad Laboratories, Hercules, CA, USA). The membranes were incubated with primary antibodies for EGFR (1:1000 dilution, Cell Signaling), EGFR (Tyr 1068) (1:1000 dilution, Cell Signaling), p42/44 (1:1000 dilution, Cell Signaling), Pp42/44 (1:1000 dilution, Cell Signaling), AKT (1:1000 dilution, Cell Signaling), pAKT (S437) (1:1000 dilution, Cell Signaling), Flag (1:1000 dilution, Cell Signaling), and GAPDH (1:1000 dilution, Cell Signaling). The membranes were incubated with horseradish peroxidase-labeled secondary antibodies (1:10,000 dilution, Cell Signaling). The signals were visualized using electrochemiluminescence detection reagents. Images of the protein bands were acquired using an ImageQuant LAS 4000 mini Biomolecular Imager and Image Quant TL software (GE Healthcare, Chicago, IL, USA).

### Statistical analysis

Data are presented as the mean ± SD of at least three independent experiments. Differences between two groups were analyzed using a paired two-tailed Student’s *t*-test. Results with *P* < 0.05 were considered statistically significant.

## Results

### Cell line characteristics

**Fig. 1:**
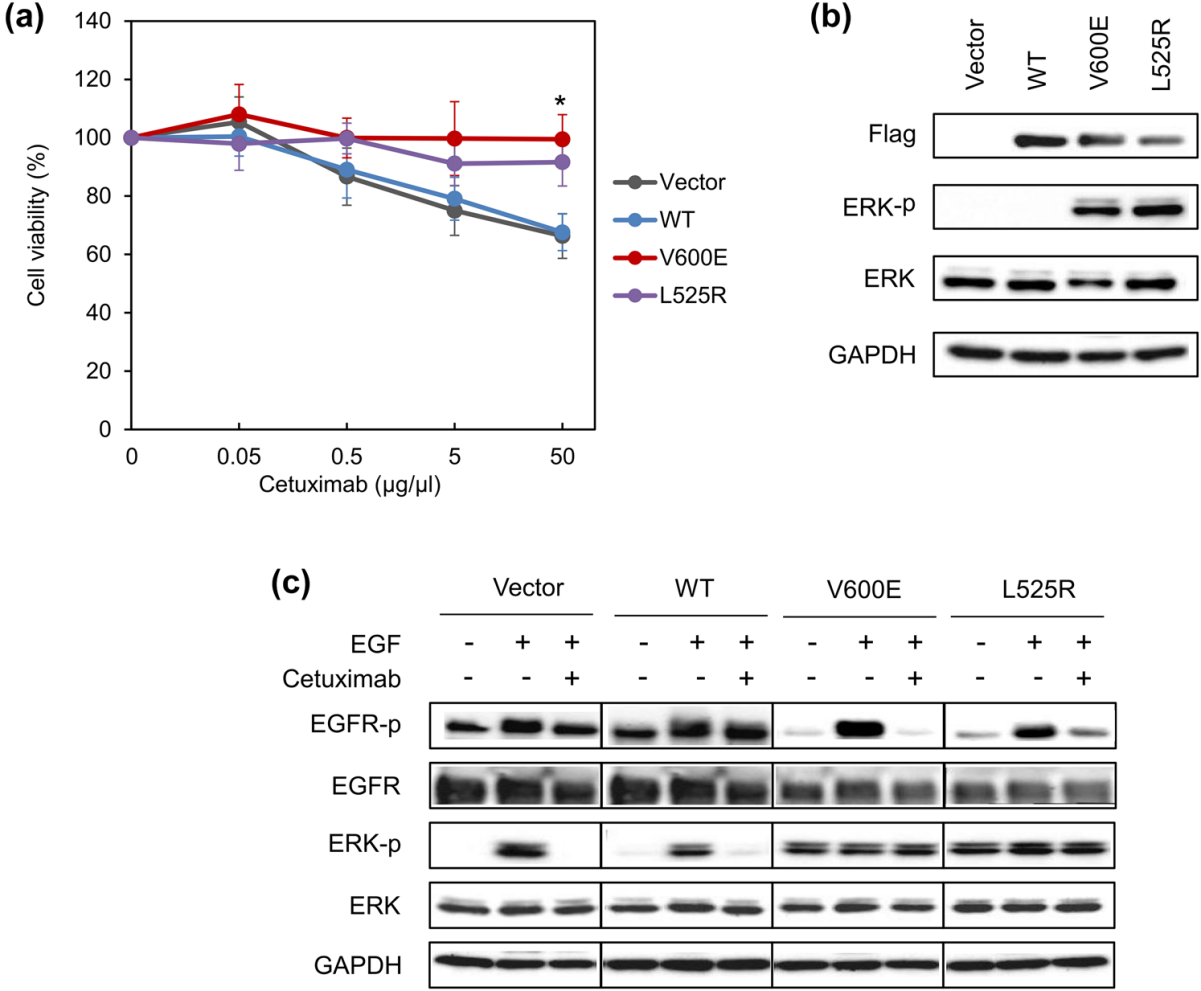
Characterization of the established *BRAF* L525R mutant cell line. **a** Effect of cetuximab on proliferation of BRAF L525R cells. Cells were treated with cetuximab for 72 h at the indicated concentrations. Cell viability was measured using Cell Counting Kit-8 (CCK-8) with the highly water-soluble tetrazolium salt WST-8. Each data point represents the mean ± SD of at least three independent experiments. *P < 0.01 indicates statistically significant difference. **b** Extracellular signal-regulated Kinase (ERK) phosphorylation in the HEK293 cells with the indicated *BRAF* mutations. **c** Effect of cetuximab on epithelial growth factor (EGF) receptor (EGFR) signaling with and without EGF stimulation. Cells were stimulated with 10 ng/mL EGF and treated or not treated with 50 μg/μL cetuximab. For western blot analysis in (**b**) and (**c**), equivalent amounts of whole cell lysates were subjected to western blot analysis to detect the indicated proteins

To characterize the biological properties of the BRAF L525R mutant, we established a culture model system. Two independent cell clones overexpressing BRAF L525R cDNA were isolated, confirmed by sequencing, and selected for individual screening. All experiments were performed using both cell clones identified, but only one set of results are shown since the reactivity of both the clones to the drugs was comparable. We first determined the growth inhibitory effect of cetuximab against the L525R-expressing cells. The cells were treated with cetuximab for 72 h at concentrations ranging from 0.05 to 50 µg/mL and cell viability was measured. Cetuximab reduced cell growth of WT cells in a dose-dependent manner but did not affect the growth of BRAF L525R or BRAF V600E cells (Fig. 1a and Fig. S1(a), *P* < 0.01). To identify the effect of L525R, basal and phosphorylated ERK protein level was evaluated by western blot analysis. ERK phosphorylation was enhanced in BRAF L525R cells to the equivalent level as in BRAF V600E cells (Fig. 1b and Fig. S1(b)), suggesting that L525R consistently activated endogenous ERK similar to V600E. Then, we evaluated the effect of the L525R on the EGFR signaling pathway. EGFR phosphorylation was observed after stimulation with 10 ng/mL exogenous EGF in Vector control, WT, V600E and L525R cells, and cetuximab treatment inhibited the EGF-induced EGFR phosphorylation. However, ERK phosphorylation caused by L525R and V600E was not inhibited by cetuximab (Fig. 1c).

### Selumetinib effectively inhibited cell proliferation and ERK phosphorylation in BRAF L525R cells

**Fig. 2:**
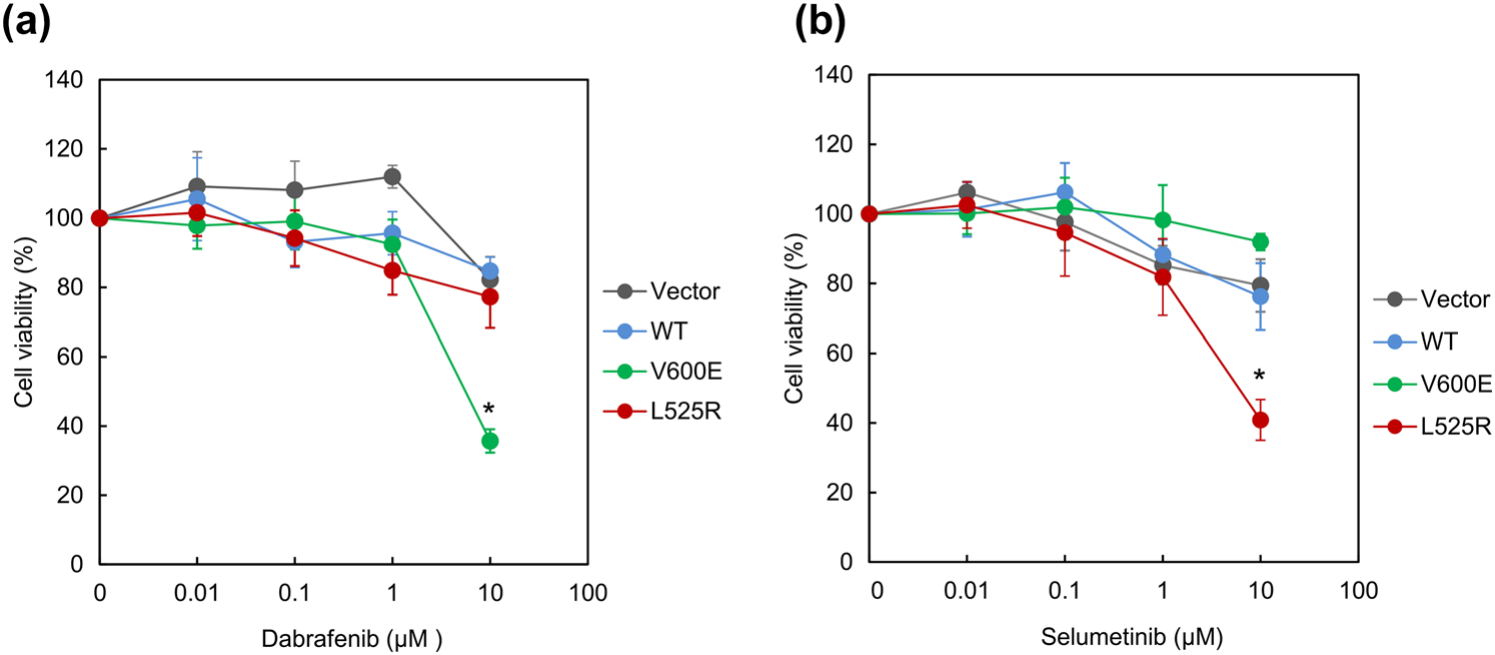
MEK inhibitor effectively inhibits extracellular signal-regulated kinase (ERK) signaling in BRAF L525R cells. Effect of dabrafenib (**a**) and selumetinib (**b**) on cell proliferation in HEK293 cells harboring the indicated *BRAF* mutations. Cells were treated with dabrafenib or selumetinib for 72 h at the indicated concentrations. Cell viability was measured using Cell Counting Kit-8 (CCK-8) assay with the highly water-soluble tetrazolium salt WST-8. Each data point represents the mean ± SD of at least three independent experiments. *P < 0.01 indicates statistically significant difference

Next, we evaluated the effect of BRAF and MEK inhibitors on L525R cells. First, we determined the proliferation of BRAF L525R and BRAF V600E cells after treatment with dabrafenib, a specific inhibitor of *BRAF* V600E [24]. Dabrafenib effectively inhibited cell proliferation of BRAF V600E cells in a dose-dependent manner; however, no significant difference was observed in BRAF L525R cells (Fig. 2a and Fig. S2(a), *P* < 0.01). We also evaluated proliferation of *BRAF* L525R and *BRAF* V600E cells after treatment with selumetinib, which is a highly specific inhibitor of MEK [25]. Selumetinib effectively inhibited cell proliferation of BRAF L525R cells in a dose-dependent manner, but no significant difference was observed in BRAF V600E cells as well as WT and vector control cells (Fig. 2b and Fig. S2(b), *P* < 0.01).

### L525R decreased AKT phosphorylation

**Fig. 3:**
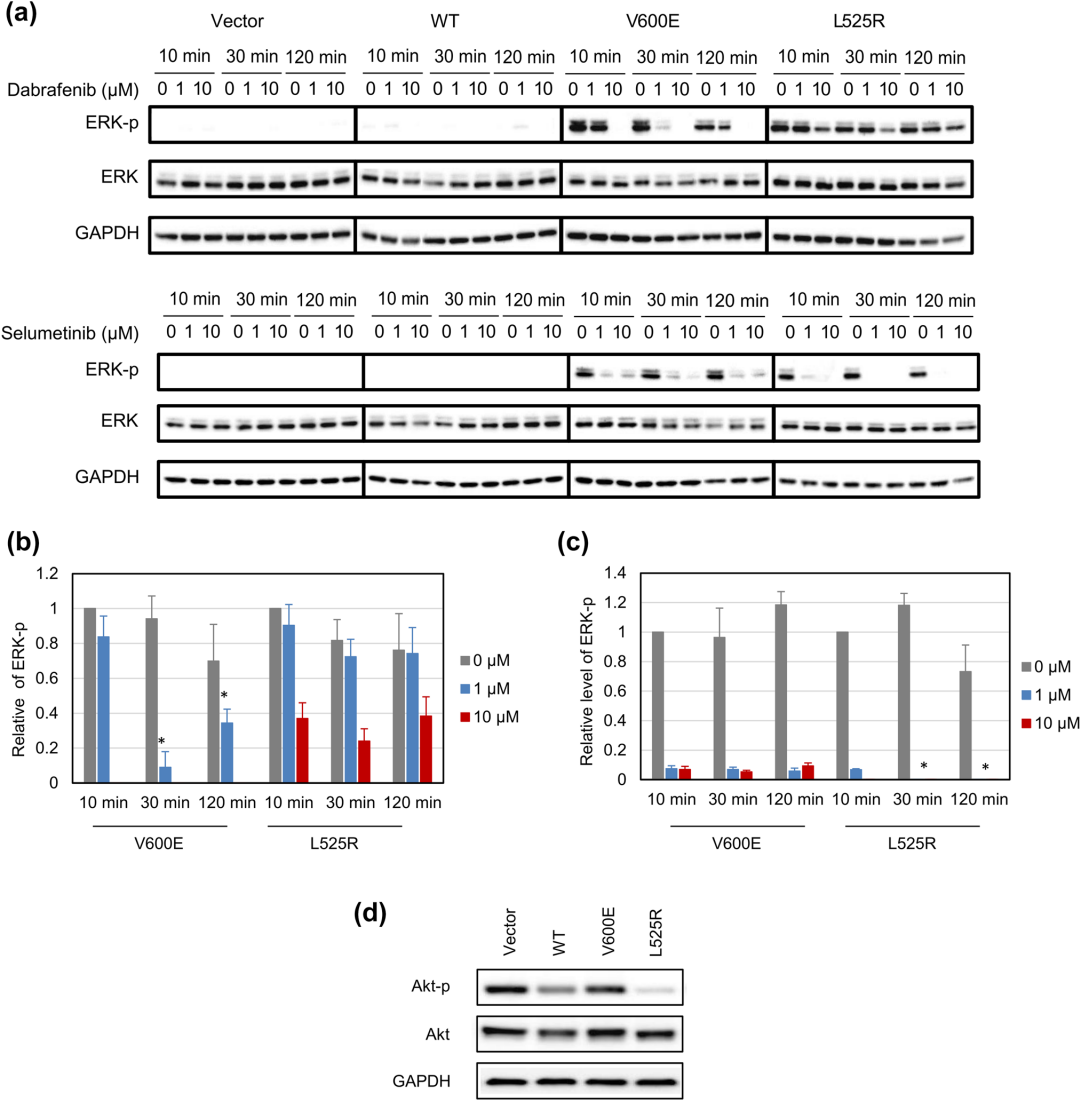
Effect of dabrafenib and selumetinib on extracellular signal-regulated Kinase (ERK) signaling. **a** Altered phosphorylation of ERK signaling proteins in HEK293 cells harboring the indicated *BRAF* mutations after treatment with the indicated inhibitors. Cells were treated with dabrafenib or selumetinib for the indicated time duration. Equivalent amounts of whole cell lysates were subjected to western blot analysis to detect the indicated proteins. **b** Relative levels of ERK-p shown in (**a**) with Dabrafenib treatment. Each data point represents the mean ± SD of at least three independent experiments. *P < 0.01 indicates statistically significant difference to selumetinib. **c** Relative levels of ERK-p shown in (**a**) with Selumetinib treatment. Each data point represents the mean ± SD of at least three independent experiments. *P < 0.01 indicates statistically significant difference to Dabrafenib. **d** Basal levels of protein kinase B (AKT) phosphorylation in HEK293 cells harboring the BRAF mutations. AKT phosphorylation were observed in untreated cells. Equivalent amounts of whole cell lysates were subjected to western blot analysis to detect the indicated proteins

To determine whether dabrafenib and selumetinib could prevent ERK phosphorylation, expression levels of phosphorylated ERK were determined by western blot analysis. Dabrafenib treatment exerted a minor inhibitory effect on ERK phosphorylation, even when BRAF L525R cells were treated with 10 µM dabrafenib; however, it inhibited ERK phosphorylation in BRAF V600E cells in a time-dependent and dose-dependent manner (Fig. 3a, b). The inhibition was the most significant after a 30-min treatment (*P* < 0.01). In comparison, treatment of BRAF L525R cells with selumetinib remarkably inhibited ERK phosphorylation in a dose-dependent manner, and to a much greater extent than observed in BRAF V600E cells (Fig. 3a, c,  *P*< 0.01). These results suggest that L525R cells are more sensitive to selumetinib than V600E cells, though both BRAF mutants equally activate ERK. Further, to characterize L525R, the phosphorylation status of another EGFR downstream target AKT was evaluated. Western blot analysis showed that basal levels of AKT phosphorylation were substantially reduced in BRAF L525R (Fig. 3d). Furthermore, whether selumetinib treatment upregulated AKT phosphorylation was analyzed. The level of AKT phosphorylation in BRAF V600E cells under selumetinib treatment was the same as that in vector control and WT cells (Fig. S3). However, the level of AKT phosphorylation in BRAF L525R cells remained low.

### AKT phosphorylation correlated with selumetinib resistance

**Fig. 4:**
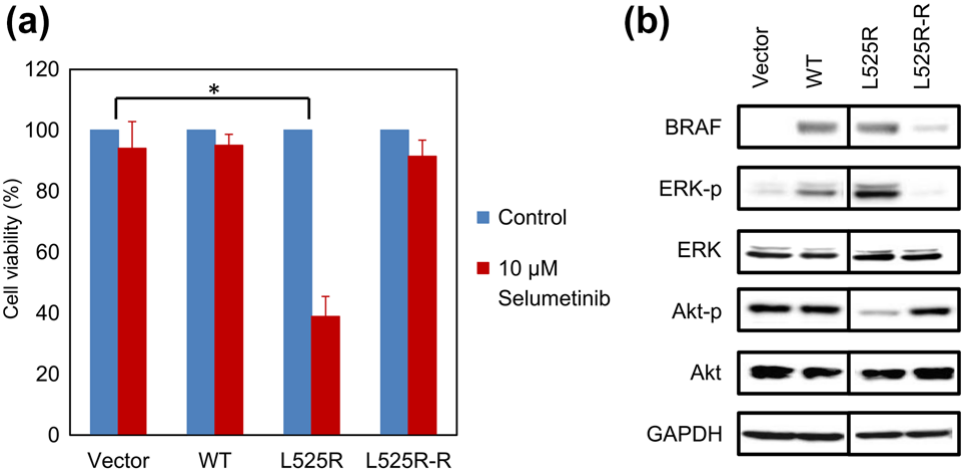
Characterization of selumetinib-resistant L525R-R cells. **a** Effect of selumetinib on proliferation of selumetinib-sensitive *BRAF* L525R cells and L525R-R cells. Cells were treated for 72 h with 10 µM selumetinib. Cell viability was measured using Cell Counting Kit-8 (CCK-8) assay with the highly water-soluble tetrazolium salt WST-8. Each data point represents the mean ± SD of at least three independent experiments. *P < 0.01 indicates statistically significant difference. **b** Decreased basal levels of extracellular signal-regulated kinase (ERK) and protein kinase B (AKT) signaling molecules in selumetinib-treated BRAF L525R and L525R-R cells. Whole cell lysates were subjected to western blot analysis to detect the indicated proteins

To confirm the association between selumetinib resistance and AKT phosphorylation, selumetinib-resistant L525R-R cells were selected by culturing BRAF L525R cells in presence of sub-lethal concentrations of selumetinib. After 2 months of selumetinib selection, two independent selumetinib-resistant clones were isolated. Results from one of the two clones are shown as the reactivity of both the clones to the drugs was comparable. The L525R-R cells were characterized with respect to cell growth and the AKT signaling pathway. Selumetinib (10 µM) strongly inhibited the proliferation of BRAF L525R cells but no such effect was observed in L525R-R cells (Fig. 4a). The basal level of ERK phosphorylation was diminished in L525R-R cells compared to that in WT cells and BRAF L525 cells, whereas AKT phosphorylation was restored to levels comparable with WT cells (Fig. 4b). The expression and phosphorylation of EGFR were also upregulated in BRAF L525R-R cells compared with those in BRAF L525R cells (Fig. S4).

### Selumetinib resistance is impaired by AKT/mTOR inhibition

**Fig. 5:**
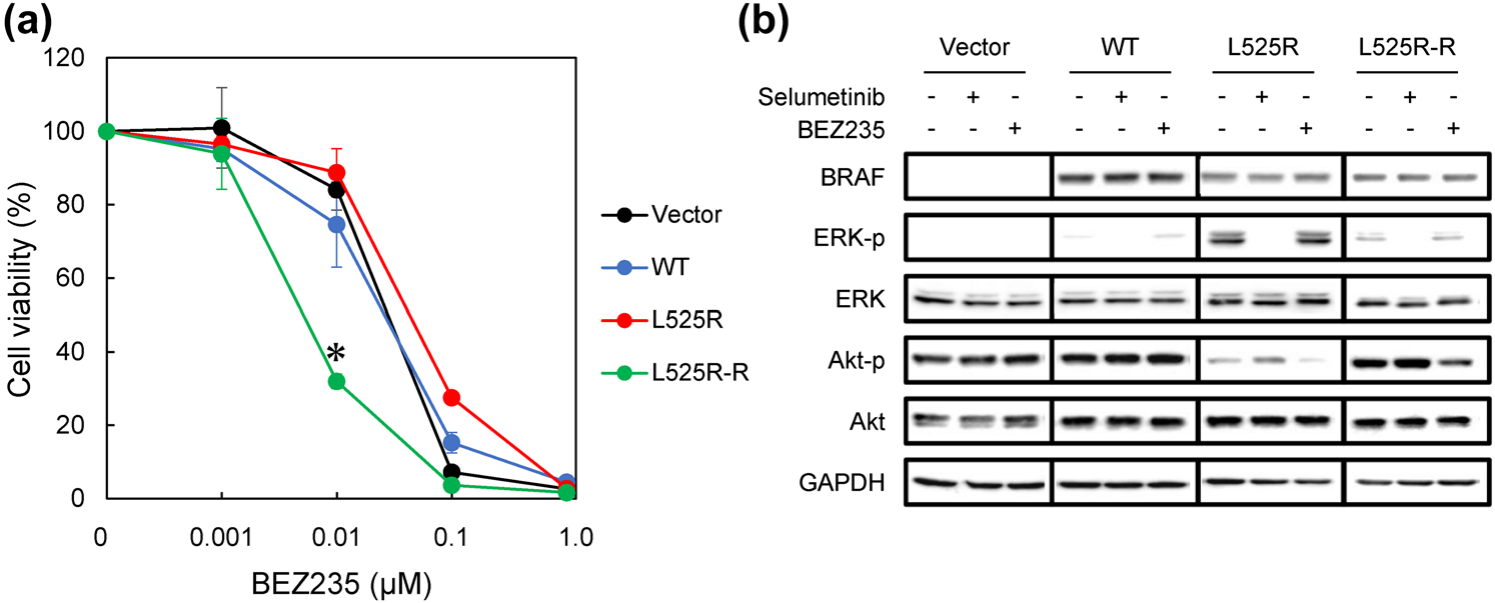
Selumetinib interacts with PI3K inhibitor BEZ235 for enhanced antiproliferative activity. **a** Effect of the dual protein kinase B (AKT)/mTOR inhibitor BEZ235 on cell proliferation. Cells were treated with BEZ 235 for 72 h at the indicated concentrations. Cell viability was measured using Cell Counting Kit-8 (CCK-8) assay with the highly water-soluble tetrazolium salt WST-8. Each data point represents the mean ± SD of at least three independent experiments. *P < 0.01 indicates statistically significant difference. **b** Effect of BEZ235 on the expression of extracellular signal-regulated kinase (ERK) and AKT signaling molecules in BRAF L525R and L525R-R cells. Cells were incubated for 30 min with 10 µM selumetinib or 0.01 µM BEZ235. Whole cell lysates were subjected to western blot analysis to detect the indicated proteins

To explore whether a combination of selumetinib and the dual AKT/mTOR inhibitor BEZ235 [26, 27] could achieve greater growth inhibition, we evaluated cell growth and ERK phosphorylation in L525R-R cells. First, we determined the sensitivity of L525R-R cells to BEZ235. We observed that treatment with BEZ235 alone inhibited cell proliferation in a dose-dependent manner in vector control, WT, the parental BRAF L525R, and BRAF L525R-R cells (Fig. 5a). The growth inhibition in L525R-R cells was achieved at a lower concentration of BEZ235 compared with that in vector control, WT, and the parental BRAF L525R cells. Moreover, cell viability was significantly lower for L525R-R cells than for BRAF L525R cells at each concentration (*P* < 0.01) (Fig. 5a and Fig. S5(a)). This indicated that L525R-R cells were more sensitive to BEZ235 compared to BRAF L525R cells. To confirm this observation, ERK and AKT signals were evaluated by western blot analysis. BEZ235 reduced AKT phosphorylation corresponding with cell growth inhibition (Fig. 5b).

The effect of combining selumetinib with BEZ235 was also evaluated using an in vitro resistant model. The combination of selumetinib and BEZ235 improved the growth inhibition, but the improving outcomes were only additive (Fig. S5(b)).

## Discussion

*BRAF* mutations are found in a wide range of cancers. Most *BRAF* mutations are activating mutations that are likely involved in oncogenesis and may therefore be attractive targets for molecular therapy. In the current study, we analyzed the function of the *BRAF* L525R mutation, which was not registered in the Catalog of Somatic Mutations in Cancer (COSMIC). This mutation was identified during our previous clinical observation study, a biomarker research for anti-EGFR monoclonal antibodies in a comprehensive cancer genomics study of patients with CRC (BREAC) [18]. A cell line harboring the *BRAF* L525R mutation had not been established before; therefore, we established a model system using recombinant EGFR-expressing HEK293 cells to analyze the in vitro response to BRAF and MEK inhibitors through the ERK and AKT signaling pathways. We demonstrated that the L525R mutation resulted in a significant increase in endogenous ERK phosphorylation, which was similar to that of the V600E mutation and constitutively stimulated the MEK/ERK signaling pathway in the absence of extracellular stimuli. We believe that this culture model system will prove useful for analyzing drug responses related to the MEK/ERK signaling pathway, even though the model did not fully represent the pathophysiological function of *BRAF* mutants.

Selumetinib is a potent and highly selective MEK inhibitor [26–28]. Selumetinib inhibited cell proliferation and ERK phosphorylation in cells harboring *BRAF* L525R mutation, while those with the *BRAF* V600E mutation were only partially inhibited, even though ERK phosphorylation was impaired. We also observed the inhibition of basal levels of AKT phosphorylation in BRAF L525R cells. Furthermore, AKT phosphorylation level in BRAF V600E cells was the same as that in vector control and WT cells, whereas the level in BRAF L525R cells remained low. Therefore, the ERK signaling pathway may be required instead of the AKT signaling pathway for the proliferation of these cells. Both ERK and AKT signaling pathways are involved in numerous biological processes. The ERK signaling pathway negatively regulates the PI3K/AKT signaling pathway in response to growth factor stimulation [29, 30]. The level of AKT phosphorylation is one of the causes of the difference in sensitivity of BRAF L525R and BRAF V600E cells to selumetinib; but, the underlying molecular mechanism remains unknown. Previous studies using melanoma cell lines reported that lower levels of phosphorylated AKT were associated with enhanced sensitivity to selumetinib [31]. In addition, the accumulating evidence suggests that compensatory activation of the PI3K/Akt signaling pathway contributes to acquired resistance to MEK inhibitors [32–34].

In BRAF L525R-R cells, the level of MEK/ERK phosphorylation decreased, whereas that of AKT phosphorylation was restored. Further, the inhibition of PI3K/mTOR with BEZ235 could overcome the acquired resistance of BRAF L525R-R cells to selumetinib. However, the expression of Flag-tagged BRAF L525R decreased in BRAF L525R cells during the acquisition of resistance to selumetinib. During prolonged selumetinib exposure, clonal selection owing to epigenetic dysregulation or genetic alteration may lead to dysregulation of *BRAF* L525R. The transcriptional upregulation and activation of receptor tyrosine kinases (RTK), including EGFR, as well as the activation of EGFR-mediated AKT signaling pathway via epigenetic deregulation results in the development of resistance to MEK and BRAF inhibitors [35–37]. These previous findings are consistent with our observations of EGFR and AKT activation in L525R-R cells.

**Fig. 6:**
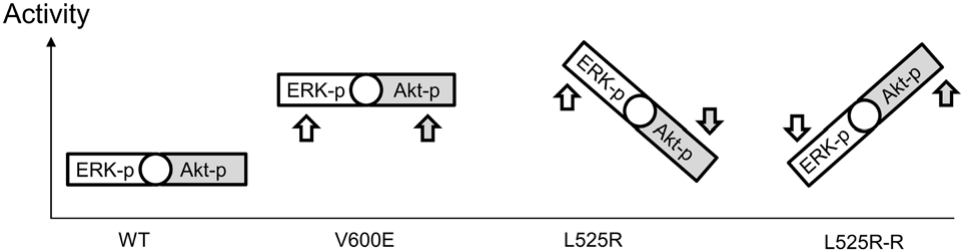
Schematic model of extracellular signal-regulated Kinase (ERK) and protein kinase B (AKT) activity in *BRAF* mutants. The *BRAF* V600E mutant shows activation of both ERK and AKT. The *BRAF* L525R mutant resulted in ERK activation with feedback regulation of AKT. In contrast, selumetinib-resistant L525R-R cells exhibited enhanced AKT activity instead of ERK inhibition

Though the precise molecular mechanisms underlying the suppression of AKT phosphorylation observed in *BRAF* L525R cells were not elucidated, *BRAF* L525R and L525R-R cells can reversibly switch between ERK and AKT signaling pathways (Fig. 6). It seems to be a strategy to maintain the homeostasis of the balance of oncogenic signals. Interestingly, this balancing was not observed in the cells expressing BRAF V600E, whose enzymatic activity was as high as BRAF L525R. The compensatory loops between ERK and AKT signaling pathways have been described in cancer therapies [38]. Further investigations on whether individual BRAF kinase mutants differently affect MEK/ERK and AKT signaling pathways are warranted. This is also clinically important to distinguish these characters for selecting the most appropriate molecular targeted drugs for patients with BRAF mutation-harboring tumors. It would be helpful to routinely analyze the activity of *BRAF* and downstream signaling pathways to evaluate the vulnerability of tumor cells.

*BRAF* is believed to be a critical cancer driver gene. The discovery of specific activating mutations in genes controlling regulatory pathways has advanced the development of mutation-specific drugs. Unfortunately, targeted therapies have led to unexpected responses in signaling networks [39]. For instance, recent analysis identified thousands of phosphorylation events orchestrated in response to MAPK blockade in *BRAF* mutated cells [40] and in mutated EGFR signaling in response to EGFR tyrosine kinase inhibitors [41, 42]. A clinical study demonstrated that a three-drug combination improves survival for patients with *BRAF* mutated CRC [43]. However, little is currently known regarding gene expression, methylation, and post-translational control of protein signaling in tumor cells of patients. Overall, the consequence of the current proteomic approach described here provides novel insights into potential anticancer therapy.

## Supplementary Information

Below is the link to the electronic supplementary material.

Supplementary file1 (DOCX 606 KB)

## Data Availability

The data that support the findings of this study are available from the corresponding author, upon reasonable request.

## Abbreviations

AKT: Protein kinase B
COSMIC: Catalog of somatic mutations in cancer
CRC: Colorectal cancer
EGFR: Epidermal growth factor receptor
ERK: Extracellular signal-regulated kinase
MEK: Mitogen-activated protein kinase
