# Supplementary material for: Regulation of MEK inhibitor selumetinib sensitivity by AKT phosphorylation in the novel BRAF L525R mutant
Source: Int J Clin Oncol. 2023 Mar 1;28(5):654–63. doi: 10.1007/s10147-023-02318-w (PMC10119053; doi:10.1007/s10147-023-02318-w)
Supplement: Supplementary file 1 — Supplementary file1 (DOCX 606 KB) [file 10147_2023_2318_MOESM1_ESM.docx]

**Supplementary figures**

***International Journal of Clinical Oncology***

**Regulation** **of** **MEK inhibitor selumetinib sensitivity by AKT phosphorylation in the novel *BRAF* L525R mutant**

Chikako Nakai^1,2^ (ORCID: 0000-0002-6201-7313), Sachiyo Mimaki^1^, Koutatsu Matsushima^2^, Eiji Shinozaki^3^, Kentaro Yamazaki^4^, Kei Muro^5^, Kensei Yamaguchi^3^, Tomohiro Nishina^6^, Satoshi Yuki^7^, Kohei Shitara^8^, Hideaki Bando^5^, Yutaka Suzuki^9^, Kiwamu Akagi^10^, Shogo Nomura^11^, Satoshi Fujii^12^, ^1^, Masaya Sugiyama^13^, Nao Nishida^13^, Masashi Mizokami^13^, Yasuhiro Koh^14^, Takuya Koshizaka^2^, Hideki Okada^2^, Yukiko Abe^2^, Atsushi Ohtsu^15^, Takayuki Yoshino^8^, and Katsuya Tsuchihara^1*^

^1^Division of Translational Informatics, Exploratory Oncology Research and Clinical Trial Center, National Cancer Center, 6-5-1 Kashiwanoha, Kashiwa, Chiba, 277-8577, Japan

^2^G&G Science Co. Ltd., 4-1-1 Misato, Matsukawamachi, Fukushima, Fukushima, 960-1242, Japan

^3^Department of Gastroenterological Chemotherapy, Cancer Institute Hospital of Japanese Foundation for Cancer Research, 3-8-31 Ariake, Koto-ku, Tokyo, 135-0063, Japan

^4^Division of Gastrointestinal Oncology, Shizuoka Cancer Center, 1007 Shimo-Nagakubo, Nagaizumi-Cho, Sunto, Shizuoka, 411-8777, Japan

^5^Department of Clinical Oncology, Aichi Cancer Center Hospital, 1-1 Kanokoden, Chikusa-ku, Nagoya, 464-8681, Japan

^6^Department of Gastrointestinal Medical Oncology, National Hospital Organization Shikoku Cancer Center, 160 Minamiumemotomachi, Matsuyama, Ehime, 791-0245, Japan

^7^Department of Gastroenterology and Hepatology, Hokkaido University Hospital, Sapporo, Japan

^8^Department of Gastroenterology and Gastrointestinal Oncology, National Cancer Center Hospital East, 6-5-1 Kashiwanoha, Kashiwa, Chiba, 277-8577, Japan

^9^Department of Computational Biology, Graduate School of Frontier Sciences, The University of Tokyo, 5-1-5 Kashiwanoha, Kashiwa, Chiba, 277-8561, Japan

^10^Division of Molecular Diagnosis and Cancer Prevention, Saitama Cancer Center, 818 Komuro, Inami-machi, Kitaadachi, Saitama, 362-0806, Japan

^11^Biostatistics Division, Center for Research and Administration and Support, National Cancer Center, 6-5-1 Kashiwanoha, Kashiwa, Chiba, 277-8577, Japan

^12^Department of Molecular Pathology, Yokohama City University School of Medicine, 3-9 Fukuura, Kanazawa-ku, Yokohama, Kanagawa, 236-0004, Japan

^13^Genome Medical Sciences Project, National Center for Global Health and Medicine, 1-7-1 Kohnodai, Ichikawa, Chiba, 272-8516, Japan

^14^Third Department of Internal Medicine, Wakayama Medical University, 811-1 Kimiidera, Wakayama, Wakayama, 641-8509, Japan

^15^National Cancer Center Hospital East, 6-5-1 Kashiwanoha, Kashiwa, Chiba, 277-8577, Japan

***Corresponding author:** Dr. Katsuya Tsuchihara

Division of Translational Informatics, Exploratory Oncology Research and Clinical Trial Center, National Cancer Center, Chiba 277-8577, Japan

Email: ktsuchih@east.ncc.go.jp


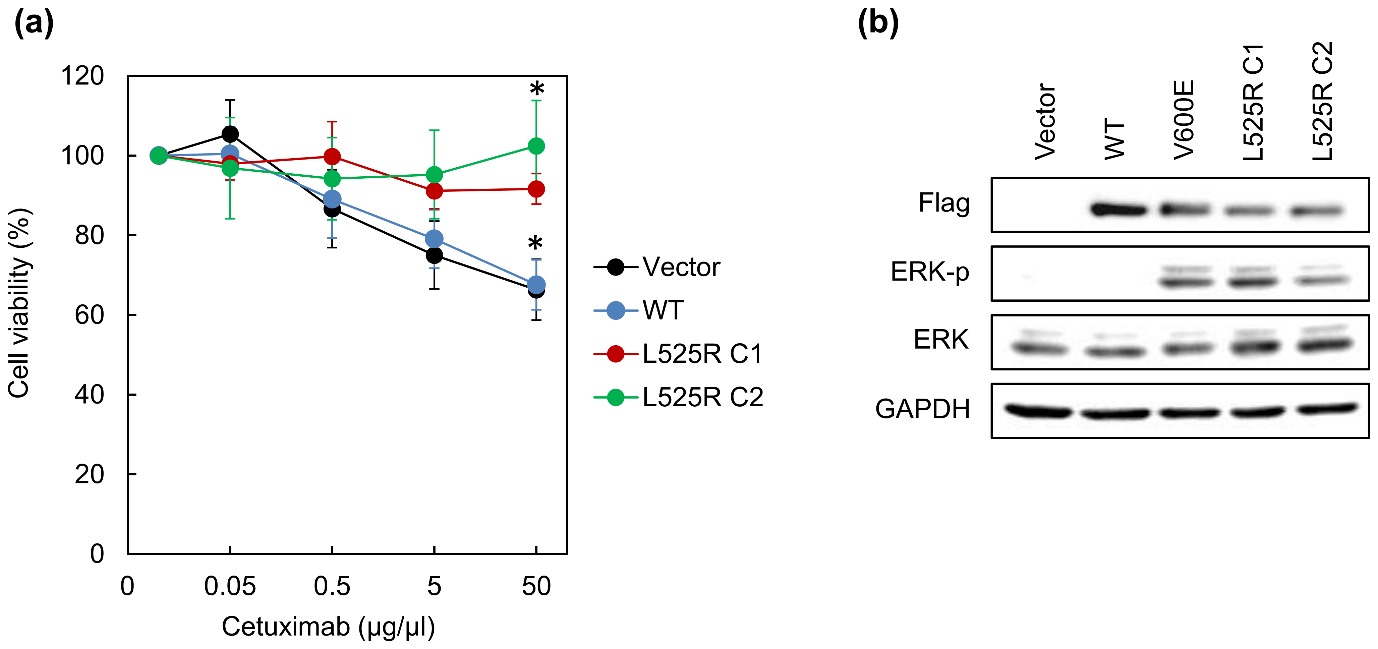


**Fig. S1 Characterization of two established clones with *BRAF* L525R mutations**

Effect of cetuximab on cell proliferation (a) and ERK phosphorylation (b). Cell viability was measured using Cell Counting Kit-8 (CCK-8) assay with the highly water-soluble tetrazolium salt WST-8. Each data point represents the mean ± SD of at least three independent experiments. *p < 0.01 indicates statistically significant difference.


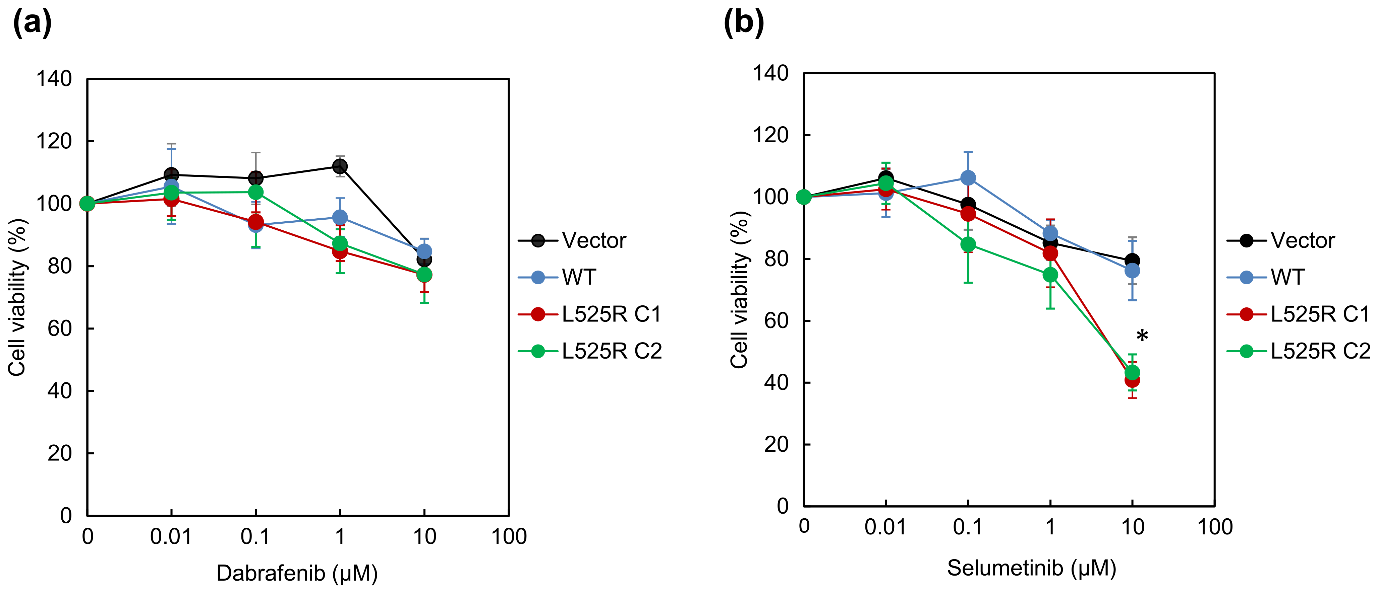


**Fig. S2 Cell viability of two *BRAF* L525R mutant clones**

Cell viability of two *BRAF* L525R mutant clones after 72 h exposure to dabrafenib (a) and selumetinib (b). Cell viability was measured using Cell Counting Kit-8 (CCK-8) assay with the highly water-soluble tetrazolium salt WST-8. Each data point represents the mean ± SD of at least three independent experiments. *p < 0.01 indicates statistically significant difference.


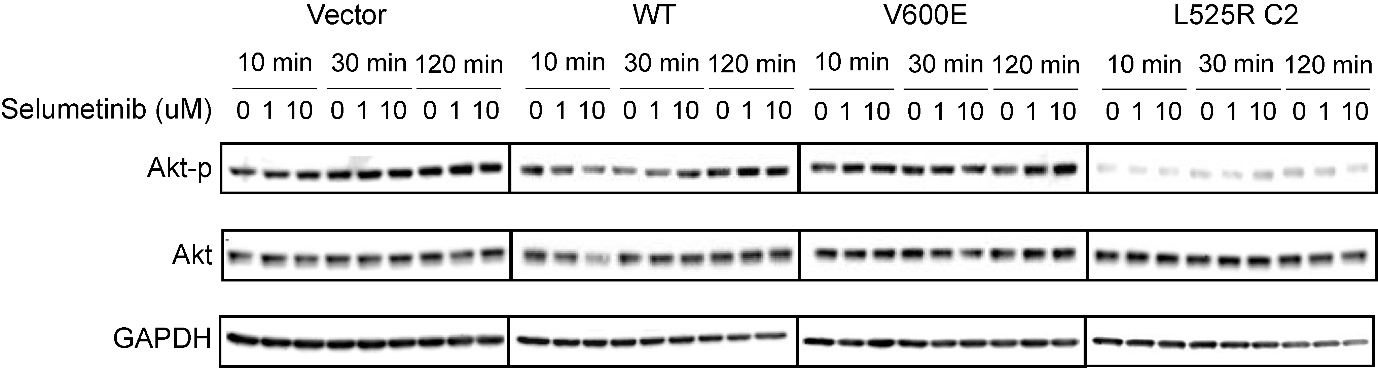


**Fig. S3 Effect of selumetinib on protein kinase B (AKT) phosphorylation**

Altered phosphorylation of AKT protein in HEK293 cells harboring the indicated *BRAF* mutations after treatment with selumetinib. Cells were treated with selumetinib for the indicated time duration. Equivalent amounts of whole cell lysates were subjected to western blot analysis to detect the indicated proteins.


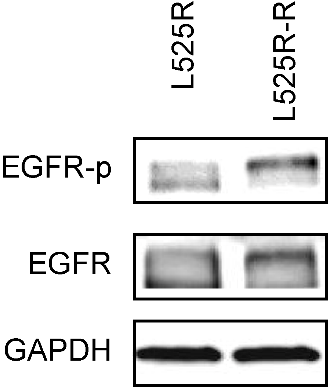


**Fig. S4 Activation of EGFR in L525R-R cells**

Expression and phosphorylation of EGFR protein in both selumetinib-sensitive HEK293 *BRAF* L525R cells and selumetinib-resistant HEK293 *BRAF* L525R-R cells.


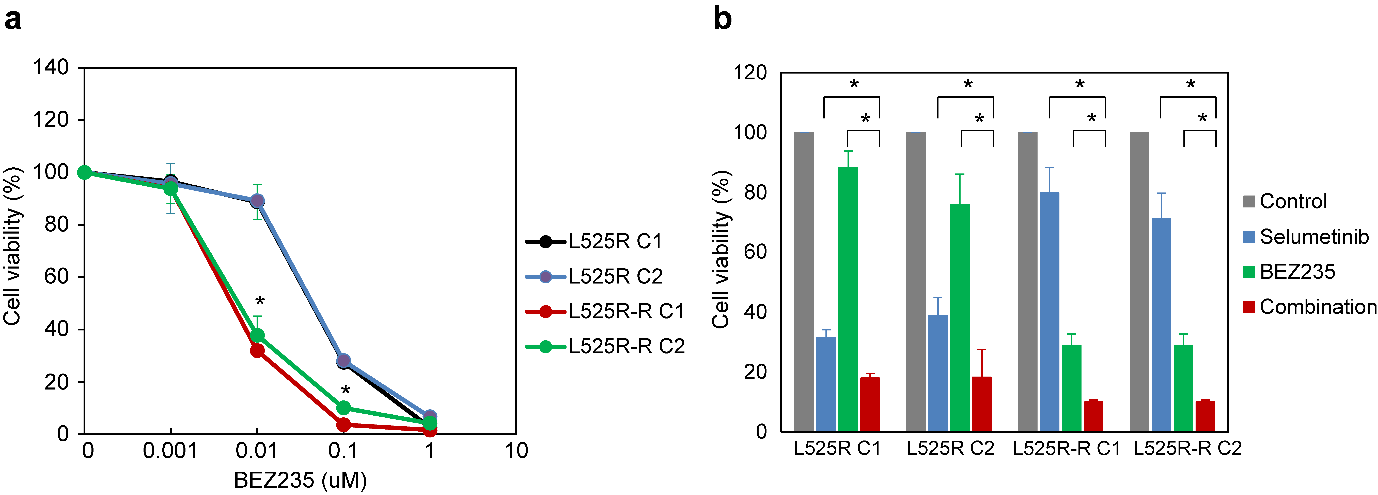


**Fig. S5 Effect of BEZ235 on cell proliferation**

Inhibition of cell growth by the dual Akt/mTOR inhibitor BEZ235 alone (a) and the combination of selumetinib and BEZ235 (b) on selumetinib-sensitive *BRAF* L525R and selumetinib-resistant *BRAF* L525R-R mutant clones. Cells were incubated for 72 h with monotherapy or the combined treatment of 10 µM selumetinib and 0.01 µM BEZ235. Cell viability was measured using Cell Counting Kit-8 (CCK-8) assay with the highly water-soluble tetrazolium salt WST-8. Each data point represents the mean ± SD of at least three independent experiments. *p < 0.01 indicates statistically significant difference.
